# Crisis management in Finnish hospital pharmacies during the COVID-19 pandemic

**DOI:** 10.1186/s12913-025-12643-7

**Published:** 2025-03-31

**Authors:** S. Latonen, A. M. Juppo, H. Seeck, M. Airaksinen

**Affiliations:** 1 Division of Pharmaceutical Chemistry and Technology, Faculty of Pharmacy, University of Helsinki, Viikinkaari 5 E (PL 56), Helsinki, 00014 Finland; 2 Department of Social Sciences, LUT University, Yliopistonkatu 34, Lappeenranta, 53850 Finland; 3 Department of Media & Communications, the London School of Economics and Political Science, Houghton Street, London, WC2A 2AE UK; 4 Division of Pharmacology and Pharmacotherapy, Faculty of Pharmacy, University of Helsinki, Viikinkaari 5 E (PL 56), Helsinki, 00014 Finland

**Keywords:** Hospital pharmacy, COVID-19, Crisis management, Finland, Pandemic preparedness

## Abstract

**Background:**

Although hospital pharmacies have played a central role in managing the COVID-19 pandemic, there is a lack of crisis management theory-based empirical research on the topic. The purpose of this study was to fill this gap in the Finnish context and identify areas for development to improve future crisis preparedness.

**Methods:**

A national cross-sectional survey was developed based on crisis management process models and sent to all hospital pharmacy heads (*n* = 21) during the second wave of the pandemic in October–November 2020. Descriptive statistics were calculated, and qualitative data from open-ended responses were studied using deductive content analysis. The results were confirmed and enriched through data triangulation with six semi-structured interviews of purposively selected hospital pharmacy heads in March–May 2021.

**Results:**

The response rate was 57% (*n* = 12). Following the onset of the pandemic, the risk perception of a crisis concerning pharmaceutical supply chain rose from 58 to 100%. A pre-existing pandemic preparedness plan was available in four (25%) pharmacies. Seven (58%) pharmacies developed a new plan. A pandemic crisis team was established in four (33%) pharmacies. Changes in internal communication and management (92%), clinical pharmacy services (67%), medicine supply (58%), procurement (42%), and pharmaceutical production operations (25%) were implemented. Collaboration with peers or other actors in the pharmaceutical supply chain increased or improved in nine (75%) hospital pharmacies, whereas in three (25%), it decreased or was unchanged. Mandatory reserve stockpiles provided a buffer for the increased need for emergency medicines. Positive and negative experiences of the pharmaceutical supply chain’s crisis response indicated an unequal distribution of medicines and crisis management-related information.

**Conclusions:**

Crisis management process models provided a holistic framework for analysing the pandemic response in hospital pharmacies. The study provided an alternative data collection approach by utilising process models in the development of the survey instrument. Preparedness of hospital pharmacies could be improved with pre-established crisis teams and plans, and data management systems providing easily accessible information to support decision-making. Developing prerequisites for coordinated information sharing and equitable distribution of medicines is essential to ensure effective crisis response, equitable medicine availability among hospitals and patient safety.

**Supplementary Information:**

The online version contains supplementary material available at 10.1186/s12913-025-12643-7.

## Introduction

The coronavirus disease 2019 (COVID-19) pandemic has challenged health systems globally, affecting supply chains and the availability of medicines in both outpatient and inpatient care [1]. Hospital pharmacies have played an important role in preventing shortages, establishing medication services to COVID-19 wards and organising vaccination logistics [2, 3]. The need for infection prevention and availability issues of medicines, disinfectants, and personal protective equipment (PPE) have necessitated swift solutions, the adaptation of processes and practices, and the management of increased workloads and stress. Thus, the public health crisis has created organisational crises, compromising the continuity of operations and the safety of employees [4, 5].

Since the beginning of the pandemic, hospital pharmacy researchers have provided valuable descriptions and takeaways of crisis management strategies in staffing, logistics, procurement, and clinical issues [6–10], the management of human resources [11], and the establishment of temporary hospitals and pharmaceutical care for COVID-19 patients [12–14]. Empirical research has covered topics such as the provision of clinical pharmacy services and pharmacist interventions [15–18], shortages, mitigation strategies and sources of information [2], the implementation of tele-pharmacy [19], home delivery services [20], and other innovative strategies during the pandemic [21]. A recent empirical survey study described crisis preparedness and response in Swiss hospital pharmacies [3]. Although hospital pharmacies played a critical role in the response to the COVID-19 pandemic, there is a lack of crisis management theory-based empirical research on this topic.

Crisis management process models represent a developmental view of crises in which a similar structure can be observed in each crisis, even though they are all unique in nature [4, 22]. The early stages of a crisis affect the latter stages; for example, a failure to respond quickly and decisively at an early stage may result in the extension of subsequent crisis stages. Developmental process models have provided useful frameworks for research on crisis management, communication, and leadership. This approach has been utilised by the World Health Organisation (WHO) and the European Centre for Disease Prevention and Control (ECDC) in their guides for national pandemic preparedness and response, as well as in Finnish national pandemic preparedness planning [23–25]. This study employs the widely accepted three-stage process model, which divides crisis management into pre-crisis, crisis, and post-crisis macro-level stages [4, 26, 27]. In *the pre-crisis stage*, efforts are focused on signal detection and risk management to prevent crises. Preparation for a crisis involves establishing crisis teams, creating crisis response plans, and conducting training. The adoption of organisational crisis management preparations depends on institutionalised practices, industry regulations, and management’s perception of risks [5, 28]. However, modest crisis management preparations may result in a false sense of security, leading to inadequate preparation and training [5]. *The crisis stage* is initiated by a crisis event, which can emerge suddenly or evolve slowly [4, 26]. The response to a crisis consists of planned and ad hoc reactions aimed at minimising damage [5]. Crisis management teams, plans, and collaboration with stakeholders have been linked to successful crisis response [5, 27, 29–31]. Crisis teams and stakeholder collaboration facilitate the continuous updating of the situation, allowing for accurate and timely actions. Finally, *the post-crisis stage* is dedicated to self-evaluation and learning from the experience, with the aim of enhancing preparedness for future crises [4, 26]. Given the complexity and unpredictability of crises, along with the numerous decisions and actions required, no organisation can respond to a crisis in a completely successful or unsuccessful manner [5, 32].

The objective of this study was to describe and investigate crisis management in Finnish hospital pharmacies during the COVID-19 pandemic using crisis management process models as a theoretical framework. Furthermore, the study sought to identify areas for development to improve future crisis preparedness in hospital pharmacies.

## Context

At the time of this study, specialised health care in Finland was provided by 21 hospital districts, including 16 regional, secondary care–level central hospitals and five tertiary care–level university hospitals [33, 34]. Each of these hospital districts houses a hospital pharmacy responsible for arranging pharmaceutical operations and services. These services include medicine supply and logistics, importing, procurement, quality and medication safety, medicine information, clinical pharmacy, and pharmaceutical production [35]. The Act on Mandatory Reserve Supplies (979/2008) obligates health-care units, the pharmaceutical industry and importers, and the National Institute for Health and Welfare to maintain crisis-related pharmaceutical stockpiles for 3–10 months. The Emergency Powers Act (1080/1991), the Health Care Act (1326/2010), and the Communicable Diseases Act (1227/2016) mandate that hospital districts and municipalities maintain preparedness and continuity management plans and implement them in their respective areas when needed.

The first wave of the COVID-19 pandemic reached Finland in March 2020, prompting the implementation of several physical distancing measures [33]. The Emergency Powers Act was brought into force for the first time since the Second World War, centralising power to the government and enabling measures to safeguard the pharmaceutical supply. During the summer of 2020, Finland transitioned from extensive restriction measures to targeted management of the epidemic. The second wave of the pandemic hit Finland in October 2020, with confirmed COVID-19 infections continuing to rise until the end of the year [36].

## Study design and methods

This cross-sectional survey study was conducted during the second wave of the pandemic, in October–November 2020. A survey was selected as the data collection method due to its advantages in infection prevention and time efficiency. It also provided a novel approach to investigating crisis management process theory, as previous studies primarily relied on interviews, documents, or media data [27]. Fifty-seven pages of interview transcripts from semi-structured interviews conducted in March–May 2021 were used for data triangulation to confirm and enrich the findings. The interview data collection has been described in detail elsewhere [37].

### Data collection

A web-based questionnaire was developed to gain a comprehensive understanding of crisis management in hospital pharmacies during the COVID-19 pandemic (see Additional file 1: Questionnaire). Crisis management process models [5, 26] provided the theoretical framework for developing the survey instrument. Existing publications on hospital pharmacies’ crisis preparedness and response were reviewed to tailor the questionnaire to the context (e.g. [16, 17, 19]). The questions were discussed, refined by the research team, and pilot tested by two individuals within the target group. Following the pilot evaluation, minor amendments were made to the wording of the three questions.

The questionnaire was structured into three sections corresponding to the pre-crisis, crisis, and post-crisis stages. The pre-crisis section aimed at assessing preparedness, covering aspects such as management’s risk perception and crisis response planning. The respondents were asked to self-evaluate their risk perceptions by answering the question, ‘How likely do you consider a crisis concerning the pharmaceutical supply chain to be?’ before and during the pandemic. The crisis section focused on the response to the crisis and the continuity of operations, touching on crisis management teams, operational changes, collaboration with stakeholders, and sources of knowledge. Finally, the post-crisis stage was designed to capture the pandemic’s impact on staff and management resilience, organisational cohesion (‘team spirit’), hospital pharmacies’ resources and finances, and self-reflection and lessons learned. The questionnaire comprised 31 questions, predominantly closed-ended (yes/no) with associated open-ended questions for elaboration, and Likert-scale queries. The online version of the questionnaire was created using the Microsoft 365 Forms web application (Microsoft Corporation, Redmond, WA, USA), and the link was disseminated to all hospital pharmacy heads (*n* = 21) via email by one of the authors. A follow-up reminder was sent two weeks later.

### Data analysis

Descriptive quantitative analysis was conducted using Microsoft 365 Excel^®^ (version 2311, Microsoft Corporation, Redmond, WA, USA). The qualitative responses from open-ended questions were grouped by question based on similarities. The answers to Q30 and Q31 were analysed together, as they both pertained to lessons learned regarding the pharmaceutical supply chain, while the responses to Q28 and Q29 were analysed together due to their focus on lessons learned from organisational crisis management (Additional file 1: Questionnaire).

Interview transcripts from six semi-structured interviews employed for data triangulation were cross-referenced with survey responses to identify divergent, corroborative, or additional quotations. Quotations related to the survey topics were primarily drawn from interview Q1, ‘*Tell us briefly about your personal experience in the hospital pharmacy’s crisis management during the COVID-19 pandemic’*, and Q22, *‘How would you improve the preparedness of the Finnish pharmaceutical supply chain for future crises?’* Other relevant parts of the interviews were also considered. The quotations were compiled using Microsoft 365 Excel^®^ software and cross-referenced with each section of the survey results.

### Research ethics

The present study followed the guidelines of the Finnish National Board on Research Integrity. Ethical approval was secured from the University of Helsinki Ethical Review Board in Humanities and Social and Behavioural Sciences (Reference number: 42/2020). The survey was conducted anonymously, with data collected, stored, and managed according to the data protection guidelines of the University of Helsinki. Informed consent was obtained from all respondents at the time of original data collection.

## Results

A total of 12 hospital pharmacy heads participated in the survey, resulting in a response rate of 57%. The participants’ demographics are detailed in Table 1.

**Table 1:** Participants’ demographics (*n* = 12)

**Characteristic**	**Description**	**n (%)**
*Job title*	Head of pharmacy	12 (100)
*Work experience as head of hospital pharmacy, years*	0–5	3 (25)
	5–10	2 (17)
	10–15	3 (25)
	15–20	2 (17)
	>20	2 (17)
*Work experience after graduation, years*	15–20	3 (25)
	>20	9 (75)
*Number of employees in the hospital pharmacy*	<40	6 (50)
	40–80	2 (17)
	>80	4 (33)

### Pre-crisis stage

**Fig. 1:**
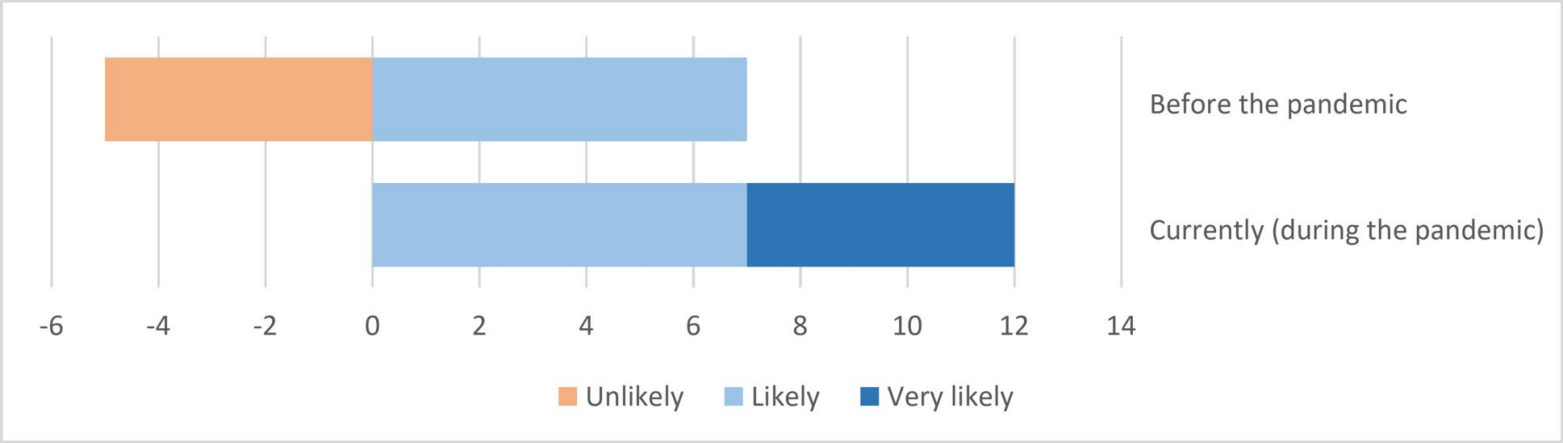
Management’s self-evaluated risk perception of a crisis concerning the pharmaceutical supply chain (evaluated on a 4-point Likert-scale). X-axis represents the number of the positive (likely-very likely) or negative (unlikely) responses. No ‘very unlikely’ responses were reported

The initial section of the questionnaire explored management’s risk perceptions and the adoption of crisis management efforts. The respondents self-evaluated the risk of a crisis affecting the pharmaceutical supply chain as ‘unlikely–likely’ on a Likert-scale before the pandemic, which shifted to ‘likely–very likely’ during the pandemic (Fig. 1).

Hospital pharmacies had pre-existing crisis response plans for a range of scenarios, including major disasters or catastrophes, pandemics, damage to the electricity system, data communication system, and water system, staff shortages, fire, issues with medicine availability or logistics, robbery, war, acute evacuation, or a general continuity plan for emergencies. These plans were communicated through training to the entire staff (*n* = 10, 83%), to the pharmaceutical staff only (*n* = 1, 8%), or solely to the head of the hospital pharmacy (*n* = 1, 8%).

Pre-existing crisis management plans were activated in response to the pandemic in four hospital pharmacies (33%). Such plans were not used in eight (67%) pharmacies, primarily due to the absence of an existing plan (*n* = 7) or their inadequacy in a real-life scenario (*n* = 1). A new crisis management plan for the COVID-19 pandemic was created in seven (58%) hospital pharmacies. Two of these pharmacies also had an existing plan, which was not sufficient for the new situation. Interview data further explained the need for a new plan: *‘Of course*,* there were those pandemic plans and others*,* but they were made with bird flu or swine flu in mind. And now*,* this was a completely different situation’ (Hospital pharmacy head*,* Interview 3).* Reasons given by respondents (*n* = 5, 42%) in open-ended questions for not creating a new crisis management plan included being part of the hospital’s pandemic plan (*n* = 3), finding the existing general pandemic plan sufficient (*n* = 1), and a lack of time (*n* = 1).

### Crisis stage

The second part of the questionnaire addressed the crisis response and continuity of operations during the COVID-19 pandemic. All hospital pharmacies reported a fast response to the pandemic, with initial actions taken either in February (*n* = 2) or March (*n* = 10) 2020. These actions related to collaboration, such as cooperation with the infection, lung, and/or intensive care units; medicine availability, including evaluation of medicine stock levels and issuing instructions and increased orders for infection and/or emergency medications; infection prevention, such as implementing visitor restrictions, encouraging remote work or other distancing measures, and placing increased orders for hand sanitiser, alcohol, and personal protective equipment; business continuity, including informing and/or training personnel or evaluating the need for additional staff; and the organisation, such as establishing a crisis team or increasing the level of preparedness. Interview data supported findings from the questionnaire:

*We quickly mapped out the arsenal of medicines likely to be used for COVID patients in collaboration with our intensive care unit and the lung department. After that*,* it was confirmed here at the pharmacy how much we had*,* and then*,* that we would probably have to buy a little more for our storage. (Hospital pharmacy head*,* Interview 6)*

**Fig. 2:**
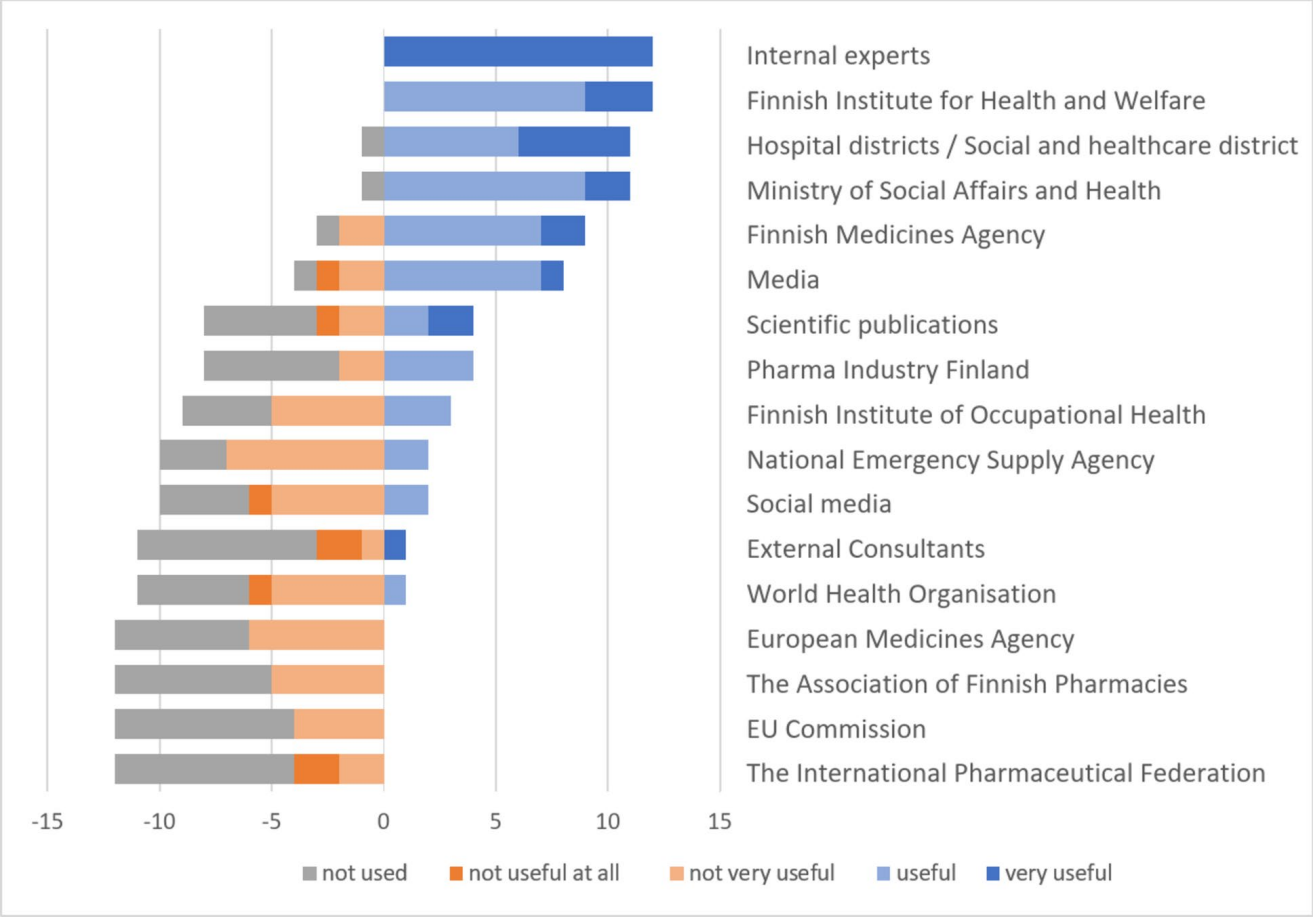
Self-evaluated usefulness of different data sources utilised in crisis management (estimated on a 5-point Likert-scale, *n* = 12). X-axis represents the number of the positive (useful-very useful) or negative (not very useful-not useful at all-not used) responses

A pandemic crisis team was set up in four (33%) hospital pharmacies. The roles of the team members were established according to their normal responsibilities (*n* = 3) or were related to the evaluation of medicine availability and storage, medicine procurement, and personnel management (*n* = 1). No crisis team was constituted in eight (67%) hospital pharmacies, where decisions were made either by the pharmacy head alone (*n* = 2) or in collaboration with other pharmacists (*n* = 6). Internal experts were perceived as the most valuable sources of information for crisis management, followed by the Finnish Institute for Health and Welfare, hospital districts, and the Ministry of Social Affairs and Health. The self-evaluated usefulness of different data sources is presented in Fig. 2.

**Fig. 3:**
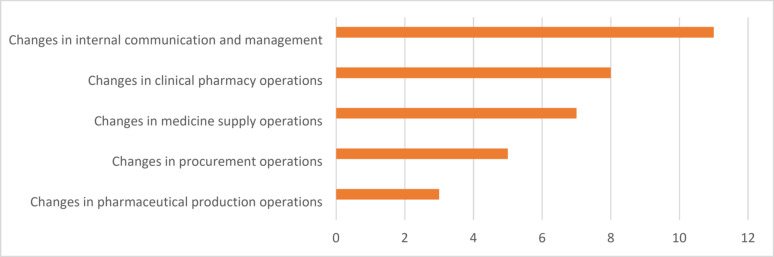
Changes in pharmacy operations during the COVID-19 pandemic (*n* = 12)

Changes in internal communication and management, clinical pharmacy, medicine supply, procurement, and pharmaceutical production operations during the pandemic are summarised in Fig. 3. Internal communication and management underwent changes in 11 (92%) hospital pharmacies, with leadership team meetings occurring daily or more frequently than before to update the situation and decide on actions. Communication shifted primarily to virtual platforms, information sharing with staff became more frequent, and the pharmacy head was included in the hospital’s pandemic response team. Adjustments in clinical pharmacy operations were noted in eight (67%) pharmacies, including reallocating pharmacists to reinforce COVID-19 and cohort wards and intensive care units, reducing movement between wards and the pharmacy, and shifting away from patient-facing roles to support medicine supply operations. Seven (58%) respondents reported changes in medicine supply operations, such as creating lists of COVID-19-related medicines, delivering these medicines to wards and home hospitals, enhancing the sharing of information on medicine availability, improving the cleaning of internal medicine delivery boxes, or employing disposable delivery boxes for infection wards. Procurement operations were altered in five (42%) pharmacies, which involved increasing stock levels for certain medicines, monitoring the consumption of pandemic-related medicines, and exploring procurement options for pandemic-related medicines or products. Pharmaceutical production operations were adapted in three (25%) pharmacies to increase the readiness to prepare pandemic-related medicines, produce pandemic-related products, such as hand disinfectant and hydrogen peroxide mouthwash, and decentralise production to two locations. Searching for alternative medications in case of availability issues was undertaken in 10 (83%) pharmacies, organising medicine lists and logistics for the COVID-19 patient ward was reported by eight (75%), and developing treatment algorithms for alternative medications was mentioned by one (8%) pharmacy. These findings were reinforced by the interviewees.

To ensure the availability of medicines during the pandemic, hospital pharmacies monitored medicine consumption and prepared to increase stocks. They increased stocks for essential medicines, agreed on necessary medicines and their alternatives with doctors, and followed instructions from government officials. To ensure the quality and safety of medicines during the pandemic, seven (58%) hospital pharmacies implemented specific measures, such as sending more guidance letters to wards, increasing the number of clinical pharmacists, and centralising pharmaceutical production expertise. Additionally, seven (58%) pharmacies redirected or augmented human resources during the pandemic. Clinical pharmacy staff were reassigned to COVID-19, infection, or intensive care wards; more pharmacists were recruited and/or trained for clinics or pharmaceutical supplies; or clinical pharmacy personnel were moved to pharmaceutical supply tasks.

Collaboration and communication with the hospital’s internal stakeholders evolved in 10 (83%) pharmacies. Collaboration intensified, with more meetings, discussions, and planning with infection, respiratory medicine, and intensive care doctors and hospital management. Moreover, collaboration shifted to virtual formats. Three (25%) respondents reported participating in the hospital’s pandemic response team. Ten (83%) pharmacies noted changes in collaboration and communication with other hospital pharmacies or entities in the pharmaceutical supply chain. Most described more frequent or regular collaboration with other hospital pharmacies (*n* = 7) or stakeholders, including government officials (*n* = 4). Collaboration topics related to medicine availability issues at the time of the survey. Collaboration with peer hospital pharmacies was praised in the interviews:

*Really good and smooth collaboration; medicines can be transferred from one place to another as needed*,* and patients can also be transferred if needed*,* so it has brought good things. (Hospital pharmacy head*,* Interview 5)*

However, in two (17%) pharmacies, collaboration or communication did not change, and in one (8%) pharmacy, it decreased. Eight (67%) respondents reported a need to develop collaboration during times of crisis. Suggested areas for development included information sharing, equitable distribution of restricted resources, encouraging open discussions rather than adhering strictly to agendas, incorporating pharmaceutical expertise in ward care, and reducing redundant work. Interview data broadened collaboration to COVID-19 vaccine storage and distribution, in which hospital pharmacies played a major role. This collaboration is depicted in more detail in [37].

### Post-crisis stage

**Fig. 4:**
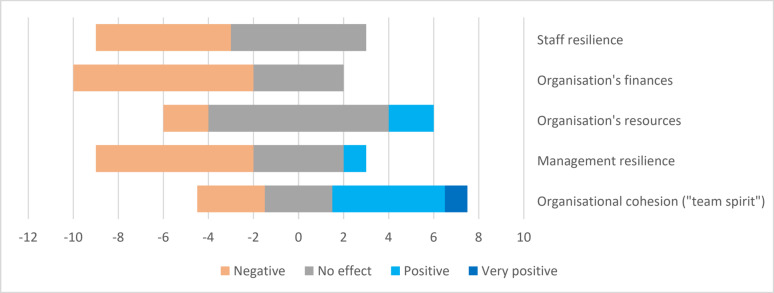
Self-evaluated impacts of the pandemic (evaluated on a 5-point Likert-scale, *n* = 12). X-axis represents the number of the positive (positive-very positive), neutral (no effect) or negative (negative) responses. Neutral responses (no effect) are positioned around zero. No ‘very negative’ responses were reported

The third part of the questionnaire focused on the post-crisis stage and consisted of an evaluation of the pandemic’s impacts, self-reflection, and lessons learned from the perspective of the hospital pharmacy and pharmaceutical supply chain. The respondents assessed the pandemic’s impacts on staff and management resilience, organisational cohesion (‘team spirit’), hospital pharmacy’s resources, and finances on a Likert- scale, as shown in Fig. 4.

Self-reflection and lessons learned from managing the crisis were solicited in Q32 and Q33 (Additional file: Questionnaire). Half of the respondents (*n* = 6) identified no areas for development at that time. Lessons learned were categorised into collaboration-related (*n* = 8), leadership-related (*n* = 7), and personnel-related (*n* = 6) themes. Collaboration-related lessons included timely and clear communication and the need for rapid meetings and solutions. Leadership-related lessons emphasised staying calm, focusing on what is most relevant, making and offering detailed, fact-based, and justifiable decisions and guidance, and taking care of one’s own resilience. Personnel-related lessons highlighted the importance of management presence, the difficulty of predicting the right actions to ensure staff sufficiency and safety, the opportunity to discuss concerns, and the training of multiple individuals in different areas to ensure backup support.

In Q30 and Q31, the respondents were asked about the successes and failures in the crisis response of the Finnish pharmaceutical supply chain (Additional file: Questionnaire). Positive factors were identified in relation to availability (*n* = 9) and collaboration (*n* = 5). Availability-related positives included the use of the mandatory reserve stockpile, generally good availability of medicines (even in intensive care), equitable distribution of medicines, and availability of hand sanitiser and alcohol. The interview data corroborated these findings and added that no serious interruptions in medical care were reported. Collaboration-related positives included collaboration with government agencies or other pharmaceutical supply chain stakeholders and sufficient information. In addition, collegial collaboration among hospital pharmacies was emphasised in the interviews.

Five (42%) pharmacy heads found no significant negative factors to mention regarding the crisis response of the Finnish pharmaceutical supply chain. However, seven (58%) respondents highlighted issues related to collaboration and communication (*n* = 4) or medicine availability (*n* = 3). Collaboration and communication-related issues included unclear or contradictory guidance on procedures or masks, data requests from the Finnish Medicines Agency during the acute crisis, absence of guidance on essential medicines to be stocked from the Finnish Medicines Agency, slow response by the National Emergency Supply Agency, and inadequate information about availability issues. These problems were echoed by two interviewees, who called for more specific guidance from authorities: *‘the availability of medicines*,* […] their limitations or restrictions*,* and the anticipation of availability problems for different groups of medicines’ (Hospital Pharmacy Head*,* Interview 3).* Medicine availability issues included shortages and the inequitable distribution of medicines to hospitals. Although the interview data reported no serious interruptions in medical care, it further elucidated challenges due to limited availability. For example, a wholesaler did not deliver hemofiltration solution to hospital according to its order, because deliveries were restricted by the pharmaceutical company holding hemofiltration solution’s marketing authorisation. According to one interviewee, a call to the pharmaceutical company revealed that the solution could be delivered based on needs assessment:

*‘… Had to call the pharmaceutical company and ask them to release [the product] from the stock balance for distribution*,* specifically for this [use]… Such a situation caused a little confusion [because] the wholesaler announced that [the product] was not available*,* but no detailed instructions were given*,* who to contact and how to act in order to get it’ (Hospital pharmacy head*,* Interview 6).*

Furthermore, the interview data further elucidated workload challenges due to availability issues, notably mentioning that the availability of hand sanitisers and their packaging materials became problematic in securing reliable substitute suppliers. According to one interviewee (Hospital pharmacy head, Interview 1), wards were instructed to return hand sanitiser pump bottles to central storage, where they were refilled from large containers and relabelled by the hospital pharmacy.

## Discussion

This study explored the crisis management process in Finnish hospital pharmacies as reported by pharmacy heads during the second wave of the COVID-19 pandemic. Almost two-thirds of all hospital pharmacy heads responded to the questionnaire. The survey data were complemented with six semi-structured interviews for data triangulation. No deviations appeared in the triangulation; instead, the survey data were supported and deepened.

According to the crisis management literature, organisational crisis management preparations depend on institutionalised practices, pharmaceutical regulations, and management’s perception of risks [5, 28]. The risk of a crisis affecting the pharmaceutical supply chain was evaluated by the respondents before and during the pandemic. Following the onset of the pandemic, the risk perception rose from 58 to 100%, indicating improved risk perception among hospital pharmacy heads. To the best of our knowledge, this is the first study reporting hospital pharmacy heads’ risk perception and how the COVID-19 has affected it. Four (25%) hospital pharmacies had a pre-existing preparedness plan, which was used during the pandemic. During the crisis, 11 (92%) pharmacies either developed a new pandemic response plan (*n* = 7), were covered by the hospital’s pandemic plan (*n* = 3), or used an existing one (*n* = 1), indicating improved preparedness during the crisis. These numbers are comparable to those of Swiss hospital pharmacies, where 24% had an existing pandemic plan before and 70% after the beginning of the pandemic [3]. Some pharmacies incorporated a new plan despite an existing one. Detailed plans tailored for a specific situation may not serve in a different crisis, however, a written plan, even one created for a different event may assists in quickly identifying feasible actions [38]. Without such, the response is less effective because more steps are required to make critical decisions. Incorporating a generic all-hazards approach to crisis plans, combined with well-chosen response strategies to known crisis scenarios is recommended instead of detailed plans, because adaptation to changing circumstances is required in each crisis [39]. Appropriate actions may vary depending on the problems that arise, crisis characteristics and organisational capacities.

Crisis teams have been linked to successful crisis outcomes, because they obtain different perspectives for issues and facilitate maintaining an updated situation picture, allowing for accurate and timely actions in crisis response [5, 31, 32]. A fast response to the pandemic was reported in all hospital pharmacies. Decisions were mostly made in pandemic crisis teams (33%) or together with other pharmacists (50%). A slightly smaller number (19%) of hospital pharmacies in Switzerland established a dedicated pandemic response team [3]. The information sources deemed most useful in crisis management were internal experts, hospital districts and government offices, such as the Finnish Institute for Health and Welfare and the Ministry of Social Affairs and Health. This finding supports previous research suggesting that health professionals prefer proximal and familiar information sources and readily accessible information [40–42]. Data management systems enabling fast access to critical information could support decision-making in crises and ordinary times.

Sharing information and resources and solving problems together with stakeholders increase the effectiveness of crisis management [27, 31, 38]. In line with earlier studies, most respondents described an increased collaboration with the hospital’s internal stakeholders, specifically with infection, respiratory medicine and intensive care doctors, and hospital management [3, 6, 9, 43]. In addition to logistical and clinical contributions to COVID-19 wards and intensive care units, the importance of pharmacy representation in the hospital crisis management team has been emphasised [9]. The present study reported that three (25%) pharmacy heads participated in the hospital pandemic response team. Previous studies have provided little information regarding collaboration with external stakeholders in the hospital pharmacy context. However, cross-sector collaboration in the pharmaceutical supply chain has been studied in the Finnish setting [37]. The present study complements previous results, showing mixed responses: collaboration and communication with other hospital pharmacies or operators of the pharmaceutical supply chain increased or improved in nine (75%) pharmacies, whereas in three (25%), it decreased or there was no change.

Mandatory reserve stockpiles provided an important buffer for the increased need for emergency medicines. The availability of medicines, information sharing with stakeholders, and equitable distribution of medicines from restricted resources were reported in both positive and negative experiences, indicating inequalities between hospital pharmacies. Similar results were described in an earlier study [37]. Equitable distribution of medicines and crisis management-related information are areas for development to ensure effective response, equitable medicine availability and patient safety in future pandemics. A national pre-established crisis management collaboration model could clarify coordinator roles, improve transparency and information sharing between government offices and pharmaceutical supply chain stakeholders [37]. Official national guidelines for managing medicine supply and avoiding misallocation, excess stockpiles or lack of medications among hospitals could be issued [44]. Interactive information infrastructure among pharmaceutical supply chain stakeholders and government offices could improve decision-making by engaging a wider exchange of knowledge and enabling equitable and fast access to correct information [45].

The present study extended crisis management process models to a new context of hospital pharmacies. Process models position learning in the post-crisis stage; however, researchers have argued that learning exists throughout the crisis cycle [46]. Moreover, the assumption that stages follow each other in a linear manner has been criticised, as they may overlap or occur simultaneously in complex and dynamic crises [47]. This was also seen in the present study, because collaboration-, leadership-, and personnel-related lessons were identified during the crisis stage. Also, process models position preparedness improving efforts in the pre-crisis stage, however, seven hospital pharmacies established new crisis management plans after the onset of the pandemic, indicating improved preparedness during the crisis stage. Despite these discrepancies, process models provided a structured and holistic approach for studying the pandemic response incorporating organisational crisis response and stakeholder relationship perspectives and allowing a look from the beginning to beyond the pandemic. In addition, the study employed an alternative data collection approach to this research area by utilising process models in the development of the survey instrument, as earlier studies mainly built on interviews and media data [27].

### Study limitations and future research avenues

Despite a satisfactory response rate of 57% and representation from hospital pharmacies of varying sizes, the total number of responses was small (*n* = 12). The survey data were supported and deepened with semi-structured interviews for data triangulation, showing no deviations. Pharmacy heads were chosen as study participants to obtain a holistic management view of the crisis management process in their organisation. However, including other relevant hospital pharmacy staff such as heads of procurement, dispensary, clinical or production departments in the study may have enriched insights into hospital pharmacies’ crisis management, particularly in cases of large hospital pharmacies. Also, focusing on a specific crisis stage could have yielded more detailed findings. Due to the focus on the early stages of the pandemic, the results reflect the first 15 months of the pandemic. This limited time frame does not capture the full spectrum of changes and adaptations that hospital pharmacies underwent during the pandemic. Moreover, participants were asked to remember events and experiences from the beginning of the pandemic, possibly leading to recall and hindsight biases, which may have influenced their responses’ accuracy and completeness.

Further research is needed on what kind of crisis preparedness efforts would best serve hospital pharmacies to provide more detailed instructions. For example, what kind of information gaps have been identified and what information is essential for hospital pharmacies to maintain core services during crises? An employee perspective would add more detailed understanding of crisis management activities and how their effectiveness were perceived. A longitudinal study is required to obtain more comprehensive picture of crisis management efforts and their changes during the pandemic, and to cover all stages of the crisis management process model. A similar data collection approach utilising process models could serve future studies, especially in the early stages of a crisis, when data collection often poses a challenge [27, 48].

## Conclusions

Crisis management process models provided a structured and holistic framework for analysing the COVID-19 pandemic response in hospital pharmacies. Process models were utilised in the development of the survey instrument, providing an alternative, structured and holistic data collection approach for studying crisis management. Hospital pharmacies navigated the pandemic through multiple adjustments in communication, collaboration, leadership, and operations. Preparedness evolved concurrently with the pandemic response. Preparedness of hospital pharmacies could be improved with pre-established crisis teams and plans, and data management systems providing easily accessible information to support decision-making. Developing prerequisites for coordinated information sharing among pharmaceutical supply chain stakeholders and equitable distribution of medicines to hospitals is essential to ensure effective crisis response, equitable medicine availability among hospitals and patient safety. Consequently, future research should explore the components of effective crisis planning and response through qualitative and longitudinal research methods within the context of hospital pharmacies.

## Supplementary Information

Additional file 1. Questionnaire.

## Data Availability

Detailed results are available from the corresponding author upon reasonable request.
