# Supplementary material for: Crisis management in Finnish hospital pharmacies during the COVID-19 pandemic
Source: BMC Health Serv Res. 2025 Mar 31;25:474. doi: 10.1186/s12913-025-12643-7 (PMC11956274; doi:10.1186/s12913-025-12643-7)
Supplement: Supplementary file 1 — Additional file 1. Questionnaire. [file 12913_2025_12643_MOESM1_ESM.docx]

**ADDITIONAL FILE 1: QUESTIONNAIRE**

**Background questions**

1. Job title
a. Executive hospital pharmacist
b. Other, please specify

2. Years of experience
a. As a hospital pharmacy head / In current position
0–5, 5–10, 10–15, 15–20,> 20 years

b. Working years after graduation
0–5, 5–10, 10–15, 15–20,> 20 years

3. How many employees are there in the hospital pharmacy?
<40, 40–80,> 80 employees

**Pre-crisis**

1. Before the COVID-19 pandemic: How likely did you consider a crisis concerning the pharmaceutical supply chain to be?

*Very likely - likely - unlikely - very unlikely*

2. Currently: How likely do you consider a crisis concerning the pharmaceutical supply chain in the future?

*Very likely - likely - unlikely - very unlikely*

3. What kind of crises does the organisation have a plan / a standard operating procedure for?

*Open answer field*

4. Who are these plans / standard operating procedures educated for? *Tick a box*

a. Head of the hospital pharmacy

b. Supervisors

c. Pharmaceutical staff

d. Whole staff

e. Other, please specify

5. Were pre-existing plans / standard operating procedures used to minimise the harm of the COVID-19 pandemic? *Yes/No*

If not: Why not? *Open answer field*

6. Did the organisation create a new plan / a standard operating procedure to minimise the harm of the COVID-19 pandemic? *Yes/No*

If not, why not? *Open answer field*

**Crisis response and business continuity during the pandemic**

7. When was the first action taken by the organisation to minimise the harm of the COVID-19 pandemic?

*MM/YYYY*

8. What was this action? *Open answer field*

9. Was a pandemic crisis team set up in the organisation? *Yes/No*

a. If yes, what are the roles of the team members? *Open answer field*

b. If not, who made the decisions to minimise the harm caused by the pandemic? *Open answer field*

10. What data sources were used to minimise the harm of the COVID-19 pandemic? Evaluate the usefulness of the data source.

*Very useful - useful - not very useful - not useful at all - not used*

a. Hospital districts / Social and healthcare district

b. Finnish Medicines Agency

c. Ministry of Social Affairs and Health

d. Department of Health and Welfare

e. National Institute of Occupational Health

f. Security of Supply Centre

g. World Health Organisation

h. The International Pharmaceutical Federation

i. European Medicines Agency

j. EU Commission

k. Internal experts

l. External Consultants

m. The Association of Finnish pharmacies

n. Pharma Industry Finland

o. Scientific publications

p. Media

q. Social media

r. Else, please specify *Open answer field*

11. What measures have been taken in the hospital pharmacy to prevent infections? *Tick a box*

a. Increasing the use of personal protective equipment

b. Improving cleaning efficiency

c. Improving hygiene: hand sanitiser, hand washing instructions

d. Precautions for incoming goods: e.g., disinfection of transport boxes

f. Dividing staff into shifts at different times

g. Remote work

h. New digital solutions or additional resources for digital solutions

i. Prohibition of visits to sales representatives / other nonhealthcare operators

j. Written instructions for visitors (only asymptomatic visitors allowed, hand and cough hygiene)

k. Instructions for staff on coronavirus, protection measures, applying for a coronavirus test, how to instruct hospital wards

l. Transfer of risk group personnel away from patient work

m. Other key measures to prevent infections, please specify *Open answer field*

12. What kind of work was done remotely? *Open answer field*

13. What kind of new digital solutions were implemented? *Open answer field*

14. Has operations of a hospital pharmacy changed during the COVID-19 pandemic? *Tick a box*

a. Changes in pharmaceutical production

b. Changes in procurement

c. Changes in supply of medicinal products

d. Changes in clinical pharmacy

e. Changes in internal communication and management

15. Please specify changes in processes. *Open answer field*

16. Additional tasks caused by the pandemic. *Tick a box*

f. Preparation of medicine lists and organisation of drug logistics in the COVID-19 patient ward

h. Searching for alternative medications in case of availability problems

i. Development of treatment algorithms for alternative medications

17. Has collaboration and communication with other hospital pharmacies or operators of the pharmaceutical supply chain changed? *Yes / No*

a. If yes, please specify *Open answer field*

18. Has the hospital pharmacy's collaboration and communication with the hospital's internal actors changed? *Yes / No*

a. If yes, please specify *Open answer field*

19. Is there a need to develop collaboration with other hospital pharmacies, operators of the pharmaceutical supply chain or hospital’s internal actors in times of crisis? *Yes / No*

a. If yes, how should cooperation be developed? *Open answer field*

20. Has the organisation taken measures to ensure availability of medicines during a pandemic?

a. Yes, measures required by the authorities

b. Yes, the measures required by the authorities and other measures, please specify *Open answer field*

21. Has the organisation taken specific measures to ensure quality of medicines, safety of medicines and / or medication safety during the pandemic? *Yes / No*

a. If yes, please specify *Open answer field*

22. Has the organisation taken measures to ensure staff resilience during a pandemic? *Yes / No*

a. If yes, please specify *Open answer field*

23. Has the organisation’s human or other resources been redirected, increased or reduced? *Yes / No*

a. If yes, please specify *Open answer field*

**Post-crisis**

24. What kind of impact has the pandemic had on staff resilience so far?

*Very negative - negative - no effect - positive - very positive*

25. What kind of impact has the pandemic had on management resilience so far?

*Very negative - negative - no effect - positive - very positive*

26. What kind of impact has the pandemic had on organisational cohesion so far (‘team spirit’)?

*Very negative - negative - no effect - positive - very positive*

27. What kind of impact has the pandemic had on public visibility of the pharmaceutical sector?

*Very negative - negative - no effect - positive - very positive*

28. What kind of impact has the pandemic had on the organisation’s resources?

*Very negative - negative - no effect - positive - very positive*

29. What kind of impact has the pandemic had on the organisation’s finances so far?

*Very negative - negative - no effect - positive - very positive*

30. What has not worked in the crisis response of the Finnish pharmaceutical supply chain? Why?

*Open answer field*

31. What has been successful in the crisis response of the Finnish pharmaceutical supply chain? Why?

*Open answer field*

32. What would you do differently in the crisis response of your own organisation?

*Open answer field*

33. What have you learned from crisis management during the COVID-19 pandemic?

*Open answer field*
